# Dual-platform screening of stripe rust resistance in advanced wheat breeding lines: integration of vegetation indices and SSR marker-based genotyping

**DOI:** 10.3389/fpls.2026.1764043

**Published:** 2026-05-14

**Authors:** Kriti Singh, Tuhina Dey, Shruthi K., Ashish Sheera, Subhash C. Kashyap, Ravinder Singh, M. K. Pandey, Simran Singh

**Affiliations:** 1 Division of Plant Breeding and Genetics, Sher-e-Kashmir University of Agricultural Sciences and Technology of Jammu, Chatha, India; 2 Division of Plant Breeding and Genetics, Shri Karan Narendra University of Agricultural Sciences and Technology, Jobner, Rajasthan, India; 3 Institute of Biotechnology, Sher-e-Kashmir University of Agricultural Sciences and Technology of Jammu, Chatha, India; 4 Department of Plant Breeding and Genetics, Punjab Agricultural University, Ludhiana, Punjab, India

**Keywords:** artificial epiphytotic, canopy temperature, chlorophyll content, normal difference vegetation index, stripe rust, wheat (*Triticum aestivum* L.)

## Abstract

Climate change-induced biotic and abiotic stresses pose a significant threat to global efforts aimed at nutritional enhancement in wheat. Stripe rust, caused by *Puccinia striiformis* f. sp. tritici (Pst), remains a major constraint to wheat production. Wheat improvement programs must now target multiple traits, particularly combining nutritional quality with durable stripe rust resistance. Integrating conventional breeding approaches with spectral vegetation indices and SSR-based genotyping offers an efficient, eco-friendly strategy for accelerating genetic gains. The present study, aimed to characterize high-micronutrient advanced wheat breeding lines developed during (2019-23) along with their parents for stripe rust resistance using both physiological and molecular tools during 2023-24. A total of 21 genotypes—comprising 13 advanced breeding lines and 8 parents—were evaluated in a Randomized Complete Block Design (RCBD) with three replications under both artificial epiphytotic and controlled conditions. Vegetation indices such as NDVI (based on NIR and IR reflectance), chlorophyll content (measured via absorbance at 650 nm and 940 nm), and canopy temperature (assessed via infrared radiation emission) were used as non-invasive indicators of plant health and stress response. Molecular analysis employed 12 SSR markers linked to known *Yr* resistance genes, among which four were found to be polymorphic but out of these four only *Barc*181 (*Yr24*) and *Xgwm*102 (*Yr16*) confirmed resistance in lines JWBL-3, JWBL-12 and JWBL-13, which also demonstrated high yield potential. Genotypes JWBL-1, JWBL-3, JWBL-4, JWBL-7, JWBL-13, and parental lines HP-25 and HP-45 exhibited lower values for Coefficient of Infection (CI) and Area Under Disease Progress Curve (AUDPC), indicating better resistance levels. These results underscore the potential of combining marker-assisted selection (MAS) with vegetation index-based physiological screening to efficiently identify and advance stripe rust-resistant, nutritionally enhanced wheat genotypes.

## Introduction

Wheat (*Triticum aestivum* L.) is a cornerstone of global food security, feeding over one-third of the world’s population. Its domestication over 8,000 years ago in the Fertile Crescent laid the foundation for its widespread adaptation and nutritional importance today (Sousa et al., 2021). In India, wheat production reached 117.507 million metric tonnes in 2024 and is projected to rise to 140 million metric tons by 2050 which underscores the crop’s significance for sustaining a growing population. Climate change, rising temperatures, erratic rainfall patterns, and soil degradation have intensified pressure on agricultural productivity (Agnihotri et al., 2020) along with hidden hunger—caused by micronutrients deficiencies of zinc, iron and selenium—remains a major global concern especially in cereal-based diets (Umar et al., 2019). These challenges are compounded by biotic stresses, predominantly stripe rust in the cool wheat growing regions caused by *Puccinia striiformis* f. sp. *tritici* (Pst). Stripe rust is one of the most devastating foliar diseases of wheat capable of causing up to 70% yield loss even under favorable conditions. Developing resistant cultivars is the most effective and sustainable approach to managing stripe rust. Traditional phenotyping methods were based on visual scoring is time-consuming, subjective and prone to environmental bias. These limitations reduce accuracy and throughput particularly when screening large, genetically diverse breeding populations (Abebe et al., 2023). Moreover, early disease detection and subtle resistance expressions are often missed which hinders effective selection. To address these limitations, modern breeding programs are increasingly integrating precision phenotyping approaches that combine advances in genomics and remote sensing. Molecular markers linked to known rust resistance genes enable marker-assisted selection (MAS), offering breeders high-resolution tools to track genetic resistance. Concurrently, multispectral sensors provide a non-destructive means to monitor crop health at scale. Vegetation indices such as the Normalized Difference Vegetation Index (NDVI), Chlorophyll content and canopy temperature have proven effective in detecting stress-related physiological changes, including early rust symptoms (Mahlein, 2016; Thirugnana Sambandham et al., 2022). The synergistic use of genomics and remote sensing remains underexplored—especially for complex, polygenic traits like adult plant resistance (APR) to stripe rust. A combined strategy of dual platform screening could significantly enhance phenotyping resolution, enabling faster, more reliable selection in breeding pipelines. This study aims to bridge this gap by evaluating advanced wheat breeding lines enriched with micronutrients for their resistance to stripe rust using an integrated precision phenotyping approach. The advanced breeding lines were developed by crossing elite genotypes from CIMMYTs HarvestPlus program with adapted commercial varieties. Field trials were conducted under both epiphytotic and disease-controlled conditions followed by integrating molecular marker and vegetation indices-based screening that captures both genetic/molecular and physiological dimensions of disease resistance. The use of NDVI for rust scoring is an advance over the manual rust scoring avoiding human bias and speed of data recording (Atanasov et al., 2025*)*. When combined with Marker assisted selection speeds varietal development process. Although vegetation indices and molecular markers have been used independently several times to study stripe rust caused by *Puccinia striiformis f.* sp. *Tritici* but their integrated application in biofortified, advanced wheat breeding lines under field epiphytotic conditions and controlled conditions still remains limited. So, this study advances existing work by implementing a dual-platform precision phenotyping approach that combines marker-based screening with multi-temporal vegetation indices. Applied to micronutrient-enriched lines developed under the HarvestPlus program of CIMMYT, this framework enables more accurate and scalable selection of disease-resistant, climate-resilient cultivars. This framework not only improves accuracy and throughput but also supports the development of climate-resilient, disease-resistant and nutritionally enhanced wheat cultivars. Our overarching hypothesis is that bridging genomics and remote sensing offers a transformative path forward for future-ready, data-driven wheat breeding programs.

## Materials and methods

### Experimental layout

The experimental material developed during 2019–23 was subjected to field layout under artificial epiphytotic and disease controlled condition during the Rabi season of 2024 using a Randomized Block Design (RBD) with three replications to assess stripe rust resistance among advanced nutrient rich wheat breeding lines. Each genotype was evaluated under two distinct blocks with artificial epiphytotic and disease controlled condition in distinct plots measuring 1m² with two rows of 2 x 0.25 meters. These blocks were spatially isolated to eliminate the risk of cross-contamination and ensure that fungicidal applications in the control. The epiphytotic block was maintained under conducive conditions for stripe rust through artificial inoculation to facilitate reliable phenotypic screening for resistance.

### Plant material

Thirteen nutritionally enriched advanced wheat breeding lines were evaluated for stripe rust resistance under artificial epiphytotic and disease-controlled conditions along eight parental genotypes which were used as source of Zn, Fe and *Yr* genes (**Tables 1**, **2**).

**Table 1:** List of advanced breeding lines used in the study.

S.no.	Advanced Breeding Lines	Pedigree
1.	JWBL-1	(HP-44X JAUW 683) X (HP-45XHD 3086)
2.	JWBL-2	(HP-44 X RSP 561)
3.	JWBL-3	(HP-45X JAUW 683) X (HP-44XHD 3086)
4.	JWBL-4	(HP-44X JAUW 683) X (HP-45XHD 3086)
5.	JWBL-5	(HP-44XRSP 561) X (HP-25XRSP 561)
6.	JWBL-6	(AGP-25XHD 3226)
7.	JWBL-7	(HP-44X JAUW 683) X (HP-45XHD 3086)
8.	JWBL-8	(HP-25XJAUW 683)
9.	JWBL-9	(AGP-25XHD 3226)
10.	JWBL-10	(HP-25XHD 3086)
11.	JWBL-11	(HD 3086XHP-25) X (RSP 561XHP-45)
12.	JWBL-12	(JAUW 683XHP-25) X (HD 3086X HP-44)
13.	JWBL-13	(HP-44XRSP 561) X (HP-44XHD 3086)

**Table 2:** Parental genotypes involved in development of advanced breeding lines.

S.No.	Parental genotypes	Pedigree	Source	Trait
1.	AGP-25	Longreach Gauntlet	IIWBR, Karnal (Australian germplasm)	*Yr18*
2.	HD 3226	GRACKLE/HD2894	IARI, New Delhi	Resistant to yellow, brown and black rust
3.	HP-44	VILLAJUAREZF2009/3/T.DIC OCCON PI94625/…	HarvestPlus (CIMMYT) through IIWBR	High zinc and Iron
4.	HP-25	KATERE//ONIX/KBIRD/6/C 80.1/3*BATAVIA//2*WBLL1/ 3/ATTILA/…	HarvestPlus (CIMMYT) through IIWBR	High zinc and Iron
5.	HP-45	KOKILA/2*VALI	HarvestPlus (CIMMYT) through IIWBR	High zinc and Iron
6.	HD 3086	DBW14/HD2733//HUW468	IARI, New Delhi	Adapted timely sown wheat
7.	RSP 561	HD2687/*Ae*. *crassa*//HD 2637	SKUAST, Jammu	Adapted timely sown and late sown wheat
8.	JAUW 683	PBW175/DBSYT421	SKUAST, Jammu	Check

### Trait evaluation

The physiological traits directly correlated to disease incidence *viz*., NDVI (Handheld GreenSeeker sensor), Chlorophyll content (SPAD-502 meter), canopy temperature (IR thermometer) and yield traits like Flag Leaf area, 1000 grain weight, plot yield were evaluated under both artificial epiphytotic and disease-controlled conditions.

### Physiological traits

#### Flag leaf area

Flag leaf area was calculated from five randomly selected flag leaves per genotype using the formula:


Leaf Area=Length×Width×0.7

The mean value per genotype was used for analysis.

#### Chlorophyll content

Chlorophyll content was measured at three stages—pre-disease, disease onset and post-disease— using a SPAD meter. SPAD readings were recorded from the middle portion of the flag leaf of five plants per plot. Values were converted to surface-based chlorophyll content (µg cm^-^²) using the equation by Cerovic et al. (2012):


(99×SPAD)/(144−SPAD)/10

#### Canopy temperature (°C)

Canopy temperature was recorded using a handheld infrared thermometer during three stages: before disease appearance, during infection and after disease progression. Readings were taken between 11:00 AM and 1:30 PM on clear days at a 30–60° angle from a height of ~60 cm. Observations were made from the sunlight side of five randomly selected plants per genotype.

#### Normalized difference vegetation index

NDVI was recorded using a GreenSeeker handheld sensor. The device calculates the ratio of NIR and Red Light Reflectance, capturing canopy greenness and ground coverage as an indicator of plant health. Measurements were taken at three stages before disease, during disease occurrence and after disease progression.

### Yield traits

#### 1000-grain weight (g)

A sample of 1000 grains per genotype was counted using an automatic seed counter and weighed using a precision electronic balance.

#### Plot yield (g/m²)

At physiological maturity, each plot was harvested and threshed separately. Grain yield was measured in grams per square meter using an electronic weighing scale.

### Pathological studies

#### Disease control

Disease-free conditions were maintained using prophylactic fungicide applications: propiconazole (25 EC) and tebuconazole (250 EC), applied before expected disease onset in late January to early February.

#### Artificial epiphytotic conditions

To ensure uniform stripe rust pressure, artificial inoculation was performed using highly susceptible wheat varieties (e.g., Agra Local, PBW 343) with high uredospore load. Infected leaves were soaked in water, supplemented with shampoo or detergent for adhesion and sprayed on target genotypes using a knapsack sprayer during early morning or late evening. Sprays were repeated until disease symptoms appeared. Two rows of susceptible infector lines were also planted to facilitate disease spread and pathotype maintenance. The pathotype information includes the use of mixture of races predominant in the region. Since this is the hotspot for the occurrence of stripe rust, major races including 110S119, 238S119, and 110S84 are prevalent.

A. Coefficient of infection (CI) (Pathan and Park, 2007).

The coefficient of infection (CI) was calculated by multiplying disease severity (%) with a constant representing host reaction, as described by Pathan and Park (2007) as per **Tables 3**, **4**.

**Table 3:** Modified Cobb’s scale for screening host response to stripe rust in field.

Reaction	Observations/Scale	Response value
No Disease	0	0.0
Resistant	R	0.2
Resistant to Moderately resistant	MRR	0.3
Moderately resistant	MR	0.4
Moderately resistant to moderately susceptible	MRMS	0.6
Moderately susceptible	MS	0.8
Moderately susceptible to susceptible	MSS	0.9
Susceptible	S	1

*The severity and response types were recorded at the same time.

**Table 4:** Scale for field recording of slow rusting parameters in wheat.

Scale	Description
TR	Traces severity of a resistant type of infection
5MS	5% severity of a moderately susceptible type of infection
10MR	10%severityofamoderatelyresistanttypeofinfection
30S or100S	30% or100% severity of a susceptible type of infection

R (Resistant) = 0.1

MR (Moderately Resistant) = 0.4

MS (Moderately Susceptible) = 0.8

S (Susceptible)=1.0

B. Coefficient of disease level (CDL) (Gupta, 1979)

Coefficient of Disease Level (CDL) integrates incidence and infection response, providing a quantitative assessment of stripe rust resistance.

It was computed using the formula:


UIV×MCI

C. UIV (Unit Incidence Value) = % incidence/100

MCI (Modified Coefficient of Infection) = Loegering’s coefficient/100

Loegering’s coefficient was estimated by multiplying disease severity with the host response value (0–1), following the method proposed by (Loegering, 1959)

CDL values range from 0 to 1, with higher values indicating increased disease susceptibility.

D. Area under disease progress curve (AUDPC)

AUDPC was calculated following the method described by Milus and Line (1986), based on disease severity over time:


$$\text{AUDPC=}\frac{{\text{N}}_{1}{\text{(X}}_{1}{\text{+X}}_{2})}{2}+\frac{{\text{N}}_{2}{\text{(X}}_{2}{\text{+X}}_{3})}{2}+\frac{{\text{N}}_{3}{(\text{X}}_{3}{\text{+X}}_{4})}{2}$$

- N_1_, N_2_, N_3_ = Time intervals between successive disease observations
- X_1_, X_2_, X_3_, X_4_ = Rust severities at respective observation times

This index reflects the cumulative disease pressure over the crop growth period.

E. Final rust severity (FRS)

Final Rust Severity (FRS) denotes the percentage of leaf area affected by stripe rust at the end of the crop season. It serves as a crucial indicator of disease impact on plant health and yield potential.

### Molecular screening using SSR primers

#### DNA extraction and genotyping

DNA extraction was done using Doyle and Doyle method and 12 SSR markers were used to assess the presence of *Yr* gene in thirteen advanced wheat breeding lines and eight parents (**Table 5**). RNase treatment was carried out at a concentration of 10 µl/ml, followed by incubation at 37 °C for 1 hour to degrade residual RNA. DNA quality and quantity were verified using 0.8% agarose gel electrophoresis. The extracted DNA was then diluted to a working concentration of 100 ng/µl using TE buffer, rendering it suitable for PCR amplification.

**Table 5:** Working parameters of SSR markers used for validation of *Yr* gene/s in wheat.

S. No.	Gene	SSR marker	Forward and reverse primer	Temp. (°C)	Reference
1.	*(Yr2)*	*Wmc364*	F: ATCACAATGCTGGCCCTAAAAC R: CAGTGCCAAAATGTCGAAAGTC	56 °C	Feng et al. (2005)
2.	*(Yr9)*	*Xgwm582*	F: AAGCACTACGAAAATATGAC R: TCTTAAGGGGTGTTATCATA	45°C	Cabuk et al. (2011)
3.	*(Yr9)*	*IB267*	F: GCAAGTAAGCAGCTTGATTTAGC R: AATGGATGTCCCGGTGAGTGG	56°C	Mago et al. (2005)
4.	(*Yr10*)	Xpsp3000	F: GCAGACCTGTGTCATTGGTC R: GATATAGTGGCAGCAGGATACG	52°C	Bariana et al. (2002)
5.	*(Yr15*)	*Xgwm273*	F: ATTGGACGGACAGATGCTTT R: AGCAGTGAGGAAGGGGATC	58°C	Revathi et al. (2010)
6.	*(Yr16*)	*Xgwm102*	F: TCTCCCATCCAACGCCTC R: TGTTGGTGGCTTGACTATTG	57°C	Agenbag et al. (2012)
7.	*(Yr18)*	*Xgwm295*	F: GTGAAGCAGACCCACAACAC R: GACGGCTGCGACGTAGAG	55°C	Bariana et al. (2010)
8.	*(Yr18)*	*Cssfr2*	F: TTGATGAAACCAGTTTTTTTTCTA R: TATGCCATTTAACATAATCATGAA	55°C	Lagudah et al. (2006)
9.	*(Yr24*)	*Barc181*	F: CGCTGGAGGGGGTAAGTCATCAC R:CGCAAATCAAGAACACGGGAGAAAGA	58°C	Wang et al. (2008)
10.	*(Yr26*)	*Barc187*	F: GTGGTATTTCAGGTGGAGTTGTTTTA R: CGGAGGAGCAGTAAGGAAGG	57°C	Wen et al. (2008)
11.	*(Yr26*)	*Xgwm498*	F: GGTGGTATGGACTATGGACACT R: TTTGCATGGAGGCACATACT	60°C	Li et al. (2006)
12.	*(Yr46)*	*CFD71*	F: CAATAAGTAGGCCGGGACAA R: TGTGCCAGTTGAGTTTGCTC	52°C	Hiebert et al. (2010)

PCR was performed to assess genetic polymorphism among wheat genotypes harboring known *Yr* resistance genes. Amplification was conducted in a total reaction volume of 15 µl using an Eppendorf Thermocycler. Each reaction mix contained 50–100 ng of genomic DNA, 8.70 µl nuclease-free water, 0.3 µl of each forward and reverse primer, 4.20 µl of 10× Taq buffer with MgCl_2_, 0.30 µl of dNTPs, and 0.2 µl of Promega GoTaq DNA polymerase. Specific thermal cycling profiles were applied for each *Yr*-linked marker, with annealing temperatures optimized according to the melting temperatures provided in the primer synthesis protocols. The PCR program included an initial denaturation at 95 °C, followed by 35 cycles of denaturation at 95 °C for 30 seconds, annealing at marker-specific temperatures for 45 seconds, and extension at 72 °C for 30 seconds. A final extension step at 72 °C was carried out to complete the amplification. The resulting PCR products were separated on a 3% agarose gel and visualized under UV light using a gel documentation system (Syngene G:BOX, USA). SSR amplification profiles were scored based on the presence (+) or absence (−) of bands. Allele sizes were estimated by comparing band migration to a 100 bp DNA ladder (Sigma Chemicals Co., USA).

### Statistical analysis

Phenotypic data was analyzed using R-studio with the R version 4.1.0 (R Core Team, 2021).

ANOVA based on a Randomized Complete Block Design (RCBD) was used to assess significant differences among genotypes.

Descriptive statistics such as mean, range, standard error, and coefficients of variation (GCV, PCV, ECV) were calculated to evaluate variability. Broad-sense heritability and genetic advance were estimated to assess the genetic potential of traits.

Independent t-tests were used for pairwise comparison of trait means.

SSR marker data were scored to evaluate presence/absence of *Yr* genes.

## Results

### Analysis of variance and components of genetic variation

The results of the analysis of variance (**Tables 6**, **7**) for 21 wheat genotypes, comprising 13 advanced breeding lines and 8 parental genotypes, revealed highly significant mean sum of squares (MSS) for treatments under both artificial epiphytotic and disease-controlled conditions for all traits, except canopy temperature before disease onset, which was significant at the 5% level. This indicates substantial genetic variation among the genotypes under both conditions, highlighting the effectiveness of advanced breeding lines in responding to disease stress and their potential contribution to yield improvement through physiological traits. The magnitude of MSS was consistently lower for canopy temperature before disease compared to NDVI and yield traits, suggesting relatively less variability in this parameter under non-stress conditions. In contrast, higher MSS for NDVI and yield traits emphasizes their usefulness as selection criteria. Notably, canopy temperature became a more discriminative trait under epiphytotic conditions, underscoring its role as a stress-responsive indicator. Replication effects were largely non-significant, indicating minimal environmental influence and confirming that the observed differences were primarily due to genetic variation. Overall, the results demonstrate that the breeding lines differ significantly in physiological responses, yield potential and disease resistance under both stress (epiphytotic) and non-stress (controlled) conditions, thereby offering opportunities to identify stable genotypes with superior photosynthetic efficiency, higher yield potential and improved resistance to disease progression.

**Table 6:** ANOVA for different vegetation indices, yield and slow rusting parameters in micronutrient introgressed advanced wheat breeding lines along with parents under artificial epiphytotic conditions.

Source of variation	d.f.	MSS														
		NDVI_i_ (BD)	NDVI_i_ (DD)	NDVI_i_ (AD)	CC_i_ (BD)	CC_i_ (DD)	CC_i_ (AD)	CT_i_ (BD)	CT_i_ (DD)	CT_i_ (AD)	FLA_i_	TGW_i_	PY_i_	AUDPC	C.I	CDL
Replication	2	0.0009	0.0005	0.0019	0.600	0.040	0.082	2.11	5.85	0.51	11.63	0.22	415.4	118332	25.305	0.000656
Treatment	20	0.0046**	0.0043**	0.0040**	2.09**	0.37**	0.63**	11.33*	6.98**	4.02**	57.24**	16.75**	8351.8 **	233179**	76.158**	0.00056378**
Error	40	0.0005	0.0003	0.0009	0.37	0.08	0.03	0.69	3.49	0.85	5.47	0.41	375.5	34427	8.025	0.000213

* and ** significant at 5% and 1% respectively; d.f., degree of freedom; NDVI_i_, Normal Difference Vegetation Index under artificial epiphytotic conditions; BD, before disease; DD, during Disease; AD, after disease; CC_i_, Chlorophyll content under artificial epiphytotic conditions; CT_i_, Canopy Temperature under artificial epiphytotic conditions; FLA, Flag Leaf Area under artificial epiphytotic conditions; TGW_i_, 1000 grain weight under artificial epiphytotic conditions; (g); PY_i_, Plot Yield; AUDPC: Area under disease progress curve; C.I, Coefficient of infection; CDL, Coefficient of Disease Level.

**Table 7:** ANOVA for various different vegetation indices and yield traits in advanced breeding lines and their parental genotypes under disease-controlled conditions.

Source of variation	d.f.						MSS						
		NDVI_c_ (BD)	NDVI_c_ (DD)	NDVI_c_ (AD)	CC_c_ (BD)	CC_c_ (DD)	CC_c_ (AD)	CT_c_ (BD)	CT_c_ (DD)	CT_c_ (AD)	FLA_c_	TGW_c_	PY_c_
Replication	2	0.0003	0.0011	0.0001	0.094	0.067	0.077	4.24	0.69	0.42	3.42	0.17	460.7
Treatment	20	0.0052^**^	0.0021^**^	0.0053^**^	1.24^**^	0.35 **	0.43^**^	3.35^*^	6.99^**^	4.10 **	29.14^**^	13.82**	23768.3**
Error	40	0.0004	0.0006	0.0006	0.12	0.06	0.06	1.41	1.36	0.45	6.76	0.53	624.6

* and ** significant at 5% and 1% respectively; d.f., degree of freedom; NDVI_c_, Normal Difference Vegetation Index under disease-controlled conditions; CC_c_, Chlorophyll content under disease-controlled conditions; CT_c_, Canopy Temperature under disease-controlled conditions; FLA_c_, Flag Leaf Area under disease-controlled conditions; TGW_c_, 1000 grain weight under disease-controlled conditions; PY_c_, Plot Yield disease-controlled condition(g). BD, before disease; DD, during Disease; AD, after disease.

### Mean performance of genotypes under stripe rust epiphytotic and disease-controlled condition

The physiological traits (Normalized Difference Vegetation Index (NDVI), chlorophyll content, canopy temperature, flag leaf area) as effected under artificial epiphytotic disease condition compared with disease-controlled condition showed a general trend of decline for NDVI, chlorophyll content, flag leaf area, TGW and yield, while canopy temperature and AUDPC exhibited an increasing trend with disease progression. Overall, disease stress reduced photosynthetic efficiency, growth and productivity compared to control.

For NDVI, a decline was observed across different stages of disease progression under epiphytotic conditions, from 0.74 ± 0.013 before, 0.67 ± 0.010 during and 0.51 ± 0.017 after disease onset as compared to control. NDVI remained higher: 0.77 ± 0.012 before, 0.72 ± 0.014 during, and 0.59 ± 0.015 after disease occurrence. The highest NDVI under epiphytotic conditions was recorded in JWBL-12 (0.82), followed by JWBL-5 (0.79) and JAUW-683 (0.78), while under control, the highest values were seen in JWBL-12 (0.85), JAUW-683 (0.84), and JWBL-10 (0.83). During disease initiation, JWBL-3 and JWBL-7 showed the highest NDVI values (0.75 and 0.72, respectively), while under control, RSP-561 and JAUW683 (both 0.75) exhibited the highest values.

Chlorophyll content showed a similar trend, with mean values before disease onset being 4.40 ± 0.354 mg/m² under epiphytotic and 4.31 ± 0.199 mg/m² under control. The highest values under diseased conditions were seen in JWBL-12 (6.01 mg/m²), JAUW-683 (5.77 mg/m²), and JAUW-6 (5.67 mg/m²), while under control, JWBL-4 (6.33 mg/m²) and JWBL-13 (5.26 mg/m²) led. During disease, the chlorophyll content ranged from 2.40 to 3.81 mg/m² under epiphytotic conditions, with JWBL-13 (3.81 mg/m²) and JWBL-3 (3.65 mg/m²) showing the highest levels, while JWBL-13 (3.99 mg/m²) and JWBL-11 (3.87 mg/m²) had the highest chlorophyll levels under control conditions.

Canopy temperature increased as the disease progressed. Before disease onset, mean canopy temperature was 12.62 ± 0.481 °C in the epiphytotic plot and 15.51 ± 0.687 °C in the control. JWBL-8 had the lowest temperature under both conditions (9.94 °C). During disease, the mean canopy temperature was higher in epiphytotic conditions (22.73 ± 1.078 °C) compared to control (20.62 ± 0.674 °C). JWBL-13 had the lowest canopy temperature during disease initiation (20.10 °C), and HP-25 showed the lowest in the control (18.07 °C).

Flag leaf area, the mean was 33.71 ± 1.350 cm² under epiphytotic conditions and 37.86 ± 1.501 cm² under control. JWBL-7 had the largest flag leaf area under disease (40.15 cm²), while JWBL-5 showed the highest under control (44.62 cm²).

The yield contributing traits 1000-grain weight (TGW) was affected by disease, with a mean of 38.38 ± 0.369 g under epiphytotic conditions and 40.10 ± 0.422 g under control. JWBL-7 had the highest TGW under disease conditions (42.23 g), and JWBL-11 had the highest under control (43 g).

Plot yield significantly differed, with a mean of 337.36 ± 11.19 g/m² under diseased conditions and 478.22 ± 14.43 g/m² under control. JWBL-3 showed the highest yield under disease (431 g/m²), while JWBL-5 had the highest yield under control (683 g/m²).

**Figure 1:**
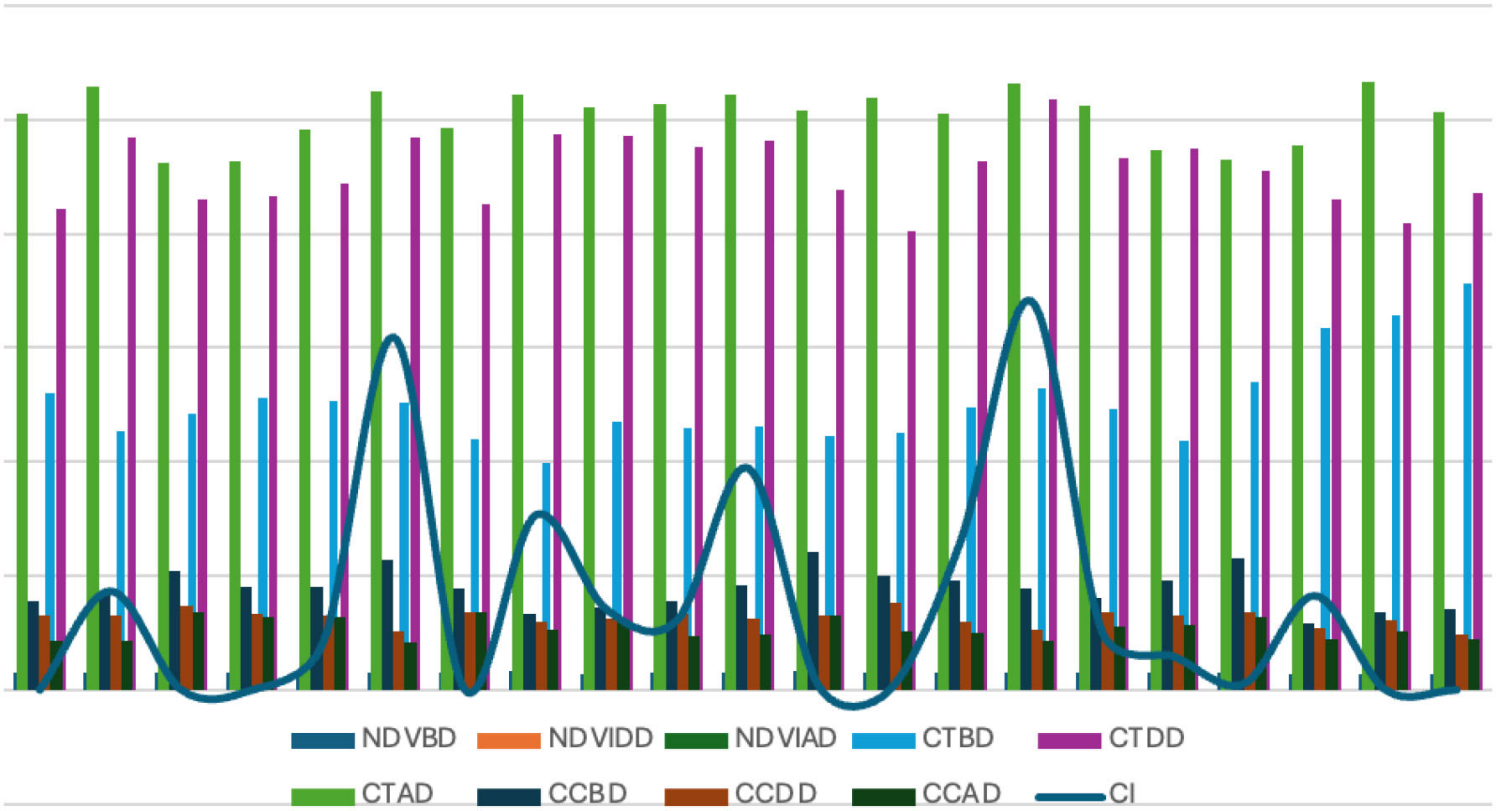
Graphical representation of vegetation indices variation at different stages of disease progression.

AUDPC an indicator of disease progression, averaged 216.45 ± 107.124 under epiphytotic conditions, with RSP-561 showing the highest disease progress (942.5). Several genotypes, including JWBL-1, JWBL-3, JWBL-4, and JWBL-7, showed zero AUDPC values, indicating no disease progression under control conditions (**Figure 1**).

### Genotypic and phenotypic evaluation of advanced wheat breeding lines and their parental genotypes

Key physiological traits exhibited high heritability, indicating strong genetic control and significant potential for improvement through selection. Traits such as NDVI, chlorophyll content, canopy temperature, flag leaf area, yield and AUDPC demonstrated considerable genetic variability making them effective predictors for resistance breeding in wheat. Under disease-inoculated conditions, NDVI displayed moderate to high heritability across disease stages—73.68% before, 81.25% during and 52.63% after disease onset. The closeness of GCV and PCV values suggests that phenotypic variance is primarily due to genotypic factors. Under control conditions, NDVI showed similar heritability levels before (80%), during (45.45%), and after (71.43%) disease, with notable genetic advance (11.39%) after disease decline, indicating potential for genetic improvement. Heritability for chlorophyll content was moderate to high at different stages of disease—60.29% before, 53.29% during, and 85.20% after disease onset. High GCV and PCV values indicate considerable genetic variability, with significant genetic advance (31.8%) during disease decline, highlighting the trait’s potential for selection. In control conditions, heritability ranged from 62.7% to 75.91%, with moderate to high genetic advance, further suggesting good potential for selection in these traits. Canopy temperature before, during, and after disease incidence showed varying heritability—83.61%, 25.04%, and 55.55%, respectively, with high GCV and PCV before and after disease indicating genetic control of the trait. FLA under disease conditions exhibited high heritability (75.95%), with moderate GCV and PCV and low genetic advance (22.12%) indicating limited potential for improvement through selection for this trait. Similar trends were observed under control.1000-grain weight had high heritability (93.03%), with close GCV (6.08) and PCV (6.30), indicating minimal environmental influence under both disease and control conditions. Plot yield showed high heritability under both disease (87.63%) and control (92.51%) conditions indicating considerable potential for genetic enhancement under disease and under control. AUDPC exhibited high genetic variability (GCV 118.69, PCV 146.32), with heritability at 65.80% and high genetic advance (198.34%) indicating significant potential for improvement, despite environmental influence during disease progression. These genetic parameters suggest that traits such as NDVI, chlorophyll content and plot yield have the highest potential for selection and improvement, making them key targets for future resistance breeding programs in wheat (**Tables 8**, **9**).

**Table 8:** Estimates of genetic parameters for vegetation indices linked to disease incidence in advanced breeding lines along with their parents under artificial epiphytotic and disease-controlled conditions.

Genetic parameters	NDVI_i_ (BD)	NDVI_c_ (BD)	NDVI_i_ (DD)	NDVI_c_ (DD)	NDVI_i_ (AD)	NDVI_c_ (AD)	CC_i_ (BD)	CCc (BD)	CC_i_ (DD)	CC_c_ (DD)	CC_i_ (AD)	CCc (AD)	CT_i_ (BD)	CTc (BD)	CT_i_ (DD)	CT_c_ (DD)	CT_i_ (AD)	CT_c_ (AD)
Range	0.67- 0.82	0.69- 0.85	0.61- 0.75	0.67- 0.79	0.46- 0.61	0.52- 0.69	2.93- 6.01	3.39- 6.33	2.40- 3.81	2.82- 4.21	2.04- 3.39	2.21- 3.62	9.94- 17.83	13.08- 17.87	20.10- 25.86	18.07- 23.28	23.09- 26.66	20.33- 25.60
Mean	0.74	0.77	0.67	0.72	0.51	0.59	4.40	4.31	3.14	3.57	2.67	3.15	12.62	15.51	22.73	20.62	25.16	23.08
GCV	5.02	5.21	5.38	3.1	6.23	6.55	17.15	14.2	9.79	8.84	16.73	11.27	14.92	5.17	4.75	6.64	4.09	4.77
PCV	5.85	5.82	5.97	4.59	8.6	7.75	22.1	16.3	13.41	11.16	18.12	13.56	16.31	9.25	8.22	8.73	5.48	5.6
Heritability (%)	73.68	80	81.25	45.45	52.63	71.43	60.29	75.91	53.29	62.7	85.2	69	83.61	92.55	25.04	57.86	55.55	52.44
Genetic Advance	0.0662	0.0737	0.0669	0.0311	0.0473	0.0674	1.2079	1.0993	0.4630	0.5151	0.8499	0.6079	3.5470	0.9255	1.1124	2.1448	1.5788	1.9368
Genetic Advance (%) Mean	8.88	9.59	9.98	4.3	9.32	11.39	27.45	25.49	14.72	14.42	31.8	19.28	28.1	5.96	4.89	10.4	6.27	10.76

NDVI_i_, Normal Difference Vegetation Index under artificial epiphytotic conditions; NDVI_C_, Normal Difference Vegetation Index under disease-controlled conditions; CC_i_, Chlorophyll content under artificial epiphytotic conditions; CC_c_, Chlorophyll content under disease-controlled conditions; CT_i_, Canopy Temperature under artificial epiphytotic conditions; CT_c_, Canopy Temperature under disease-controlled conditions; BD, before disease; DD, during Disease; AD, after disease.

**Table 9:** Estimates of genetic parameters for different yield traits and slow rusting parameters in advanced breeding lines along their parents under both artificial epiphytotic and disease-controlled conditions.

Genetic parameters	FLA_i_	FLA_c_	TGW_i_	TGW_c_	PY_i_	PY_c_	AUDPC	C.I	CDL
Range	23.18-40.15	33.03-44.62	33.07-42.23	35.66-43.00	240.33-430.33	320-683	0.00-942.50	0.00-17.07	0.000-0.074
Mean	33.71	37.86	38.38	40.10	337.36	478.22	216.86	3.7168	0.0073
GCV	12.32	7.21	6.08	5.25	15.28	18.37	118.69	128.22	137.65
PCV	14.14	9.96	6.3	5.55	16.33	19.09	146.32	149.16	238.41
Heritability (%)	75.95	72.45	93.03	89.26	87.63	92.51	65.80	73.89	33.33
Genetic Advance	7.4584	4.0735	4.6379	4.0964	99.4314	174.0265	395.1155	8.4387	0.0119
Genetic Advance (%) Mean	22.12	21.01	12.08	10.21	29.47	36.39	198.34	227.04	163.8

FLA, Flag Leaf Area under artificial epiphytotic conditions; FLA, Flag Leaf Area under disease-controlled conditions; TGW, 1000 grain weight under artificial epiphytotic conditions; (g); TGW, 1000 grain weight under disease-controlled conditions PY, Plot Yield under artificial epiphytotic condition; PY, Plot Yield disease-controlled condition(g). BD, before disease; DD, during Disease; AD, after disease; AUDPC, Area under disease progress curve; C.I, Coefficient of infection; CDL, Coefficient of Disease Level for artificial epiphytotic.

### Independent t-test for comparative assessment to genotypes under epiphytotic and diseased conditions

The independent *t*-test (**Table 10**) was employed to compare vegetation indices, slow rusting parameters, and yield traits under artificial epiphytotic and disease-controlled conditions. Levene’s test for homogeneity of variances indicated that equal variances could be assumed for most traits, except for NDVI during disease and the slow rusting parameters (AUDPC, CDL, CI), where unequal variance was considered. Before disease onset, no significant differences were observed for NDVI and chlorophyll content between the two conditions, indicating that the physiological status of genotypes was largely comparable prior to stress imposition. In contrast, canopy temperature already showed significant variation before disease, suggesting inherent genotypic differences in stomatal regulation and transpiration efficiency even under non-stress conditions. With the onset of disease, significant differences (p< 0.05) were observed between epiphytotic and control conditions for NDVI, chlorophyll content, canopy temperature, yield traits, and slow rusting parameters. NDVI exhibited a marked reduction during and after disease, highlighting the decline in photosynthetic activity and canopy greenness. Similarly, chlorophyll content showed a significant decrease under epiphytotic conditions, indicating disease-driven chlorophyll degradation and loss of photosynthetic pigments. In contrast, canopy temperature increased significantly during and after disease, reflecting reduced transpiration and impaired stomatal conductance in diseased plants. Yield-related traits, including flag leaf area, thousand-grain weight, and plot yield, were significantly reduced under epiphytotic conditions, demonstrating the adverse impact of disease stress on assimilate partitioning and grain filling. Meanwhile, slow rusting parameters—AUDPC, CDL and CI—were significantly elevated under epiphytotic conditions, validating the effectiveness of artificial disease establishment and enabling the clear separation of resistant and susceptible genotypes. Overall, the *t*-test results confirm that genotypes differed significantly in their physiological and yield responses under stress and non-stress conditions. The lack of variation in NDVI and chlorophyll content before disease but strong divergence during and after disease highlights their reliability as stress-inducible traits. Conversely, canopy temperature showed discriminative power even before disease onset and became more pronounced under stress. Together, these findings emphasize NDVI, chlorophyll content, canopy temperature, yield traits, and slow rusting parameters as robust indicators for screening wheat breeding lines under artificial epiphytotic environments.

**Table 10:** Independent t-test comparing vegetation indices, yield and slow rusting parameters under artificial epiphytotic and controlled conditions.

		Levene’s test for equality of variances					T-test for equality of means			
		F	Sig.	T	d.f.	Sig. (2-tailed)	Mean difference	Std. error difference	95% Confidence interval of difference	
									Lower	Upper
NDVI_BD_	Equal variances assumed	0.008	0.929	-1.827	40	0.075	-0.02286	0.01251	-0.04815	0.00243
	Equal variances not assumed			-1.827	39.826	0.075	-0.02286	0.01251	-0.04815	0.00244
NDVI_DD_	Equal variances assumed	5.367	0.026	-5.041	40	0.000	-0.05238	0.01039	-0.07338	-0.03138
	Equal variances not assumed			-5.041	36.537	0.000	-0.05238	0.01039	-0.07344	-0.03132
NDVI_AD_	Equal variances assumed	0.477	0.494	-7.022	40	0.000	-0.08429	0.01200	-0.10854	-0.06003
	Equal variances not assumed			-7.022	39.419	0.000	-0.08429	0.01200	-0.10855	-0.06002
CC_BD_	Equal variances assumed	1.934	0.172	0.381	40	0.705	0.08762	0.23002	-0.37726	0.55250
	Equal variances not assumed			0.381	37.599	0.705	0.08762	0.23002	-0.37819	0.55343
CC_DD_	Equal variances assumed	0.017	0.898	-3.991	40	0.000	-0.42857	0.10740	-0.64563	-0.21151
	Equal variances not assumed			-3.991	39.991	0.000	-0.42857	0.10740	-0.64563	-0.21151
CC_AD_	Equal variances assumed	1.928	0.173	-3.687	40	0.001	-0.48095	0.13043	-0.74457	-0.21733
	Equal variances not assumed			-3.687	38.652	0.001	-0.48095	0.13043	-0.74486	-0.21705
CT_BD_	Equal variances assumed	3.555	0.067	-5.988	40	0.000	-2.89048	0.48270	-3.86604	-1.91491
	Equal variances not assumed			-5.988	30.869	0.000	-2.89048	0.48270	-3.87511	-1.90584
CT_DD_	Equal variances assumed	0.134	0.716	4.476	40	0.000	2.10762	0.47084	1.15602	3.05922
	Equal variances not assumed			4.476	40.000	0.000	2.10762	0.47084	1.15602	3.05922
CT_S_	Equal variances assumed	0.166	0.686	5.789	40	0.000	2.07857	0.35906	1.35288	2.80426
	Equal variances not assumed			5.789	39.996	0.000	2.07857	0.35906	1.35288	2.80426
FLA	Equal variances assumed	1.463	0.234	-3.539	40	0.001	-4.14429	1.17097	-6.51090	-1.77767
	Equal variances not assumed			-3.539	36.177	0.001	-4.14429	1.17097	-6.51871	-1.76986
TGW	Equal variances assumed	0.216	0.645	-2.467	40	0.018	-1.71857	0.69660	-3.12645	-0.31070
	Equal variances not assumed			-2.467	39.640	0.018	-1.71857	0.69660	-3.12685	-0.31030
PY	Equal variances assumed	3.130	0.084	-6.239	40	0.000	-140.77952	22.56587	-186.38684	-95.17220
	Equal variances not assumed			-6.239	32.527	0.000	-140.77952	22.56587	-186.71550	-94.84354
AUDPC	Equal variances assumed	24.157	0.000	3.565	40	0.001	216.85714	60.83797	93.89902	339.81527
	Equal variances not assumed			3.565	20.000	0.002	216.85714	60.83797	89.95136	343.76292
CDL	Equal variances assumed	20.328	0.000	2.683	40	0.011	.012905	0.004811	0.003182	0.022627
	Equal variances not assumed			2.683	20.000	0.014	.012905	0.004811	0.002870	0.022940
CI	Equal variances assumed	26.335	0.000	3.380	40	0.002	3.716667	1.099500	1.494493	5.938840
	Equal variances not assumed			3.380	20.000	0.003	3.716667	1.099500	1.423149	6.010184

BD, Before Disease; DD, During Disease and AD, After Disease.

### Field-based stripe rust screening in micronutrient introgressed advanced wheat breeding lines and their parental genotypes

**Figure 2:**
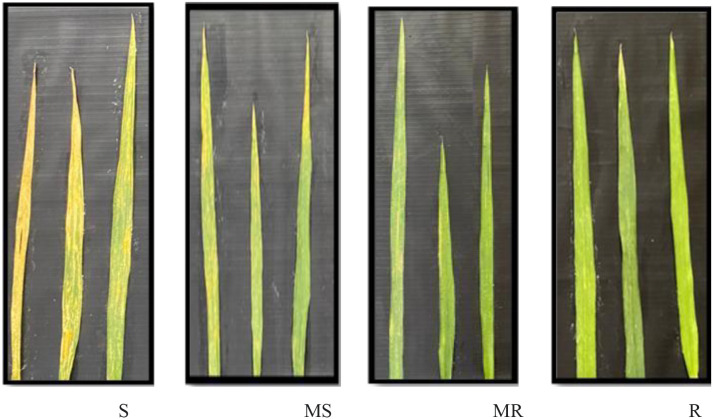
Representative array of stripe rust incidence on wheat leaves.

**Figure 3:**
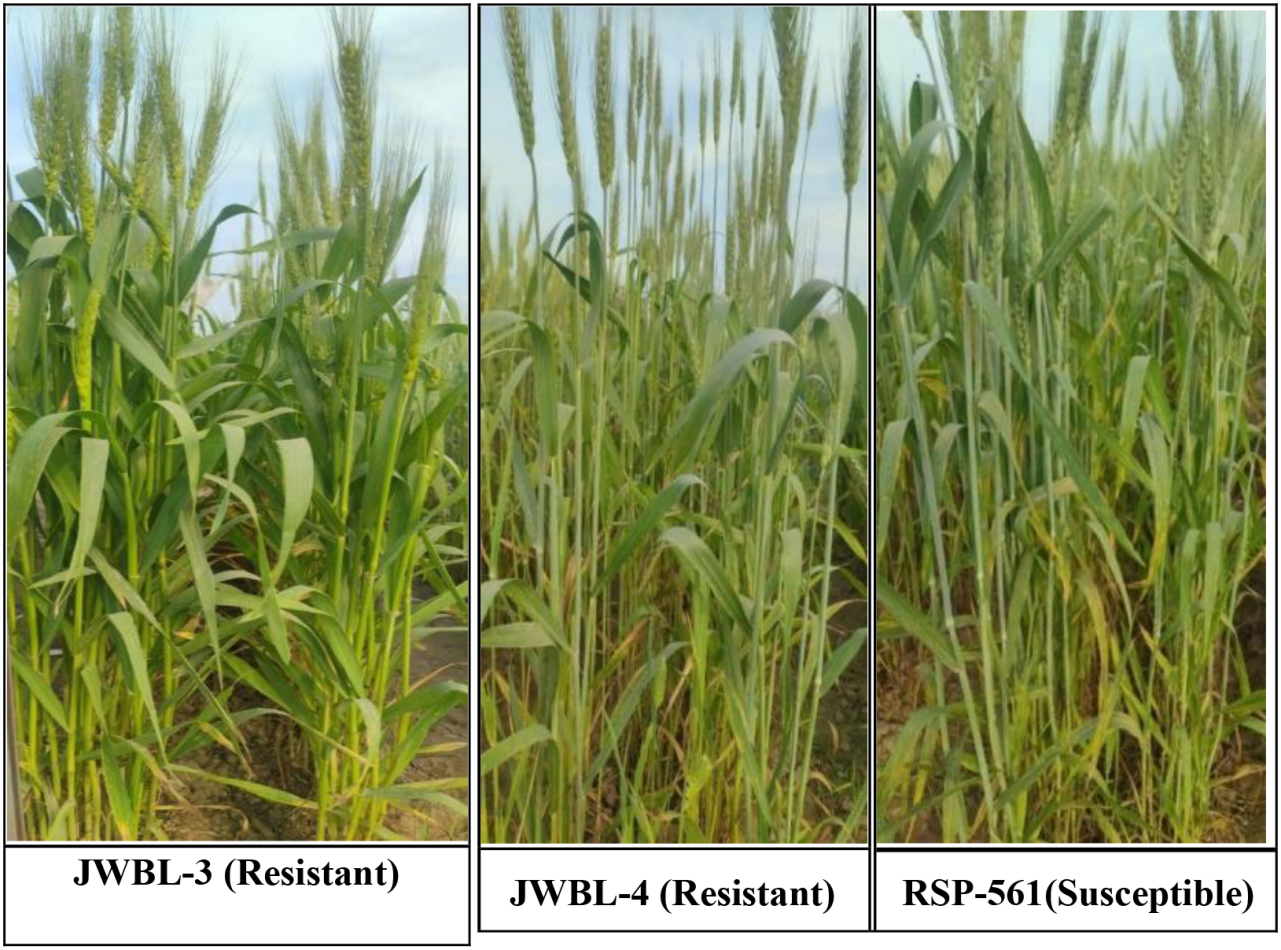
Disease reaction of JWBL-3, JWBL-4 and RSP-561 under field conditions.

The genotypes were scored based on their response to stripe rust as Resistant (R), Moderately Resistant (MR), Moderately Resistant to Moderately Susceptible (MRMS), and Moderately Susceptible to Susceptible (MSS), The breeding lines developed could be classified into all categories with Resistant genotypes showing no disease incidence *viz.*, JWBL-1, JWBL-3, JWBL-4, JWBL-7, JWBL-12, JWBL-13, JAUW-683, HP25, and HP-45. Parental genotypes Longreach Gauntlet was the only genotype exhibiting moderate resistance. Genotypes such as JWBL-5 and HD-3226 displayed MRMS reactions, while JWBL-10 and JWBL-11 ranged from MRMS to MSS. JWBL-2, HD-3086, and HP-44 exhibited MSS to MS reactions. The most susceptible lines, including JWBL-6, RSP-561, and JWBL-8, showed MS to S reactions. Slow rusting parameters were assessed through Final Rust Severity (FRS), Coefficient of Infection (CI), Coefficient of Disease Level (CDL), and Area Under the Disease Progress Curve (AUDPC). Genotypes *viz.*, JWBL-1, JWBL-3, JWBL-4, JWBL-7, JWBL-13, HP-25, and HP-45 demonstrated high resistance with low FRS, CI, CDL, and AUDPC values indicating minimal disease impact and slower rust progression. In contrast, RSP-561, JWBL-6, and JWBL-11 exhibited high susceptibility as reflected through higher CI, CDL, and AUDPC values indicating aggressive disease progression and greater severity (**Table 11**; **Figures 2**, **3**).

**Table 11:** Field based slow rusting parameters in parental and advanced breeding lines.

S. No.	Genotypes	FRS	Average CI	Average CDL	Average AUDPC
1.	JWBL-1	TMR	0.00	0.000	0
2.	JWBL-2	20MS	4.33	0.012	220
3.	JWBL-3	0	0.00	0.000	0
4.	JWBL-4	0	0.00	0.000	0
5.	JWBL-5	10MRMS	2.07	0.003	117.5
6.	JWBL-6	40S	15.48	0.074	888.5
7.	JWBL-7	TR	0.00	0.000	0
8.	JWBL-8	20S	7.67	0.029	425
9.	JWBL-9	10S	3.53	0.010	187.5
10.	JWBL-10	15MSS	3.07	0.004	170
11.	JWBL-11	40MSS	9.73	0.040	517.5
12.	JWBL-12	10R	0.16	0.000	7
13.	JWBL-13	TR	0.00	0.000	0
14.	HD-3086	15MS	6.53	0.009	407.5
15.	RSP-561	60S	17.07	0.069	942.5
16.	Longreach Gauntlet	20MR	2.53	0.006	150
17.	HD-3226	15MRMS	1.48	0.003	78.5
18.	JAUW-683	10R	0.27	0.000	15
19.	HP-44	20MS	4.13	0.012	230
20.	HP-25	TR	0.00	0.000	0
21.	HP-45	0	0.00	0.000	0

CI, Coefficient of Infection; CDL, Coefficient of Disease Level; AUDPC, Area under disease progress curve.

**Table 12:** Correlation between vegetation indices and slow rusting parameters before onset of disease under artificial epiphytotic condition.

	NDVI_i (BD)_	CC_i (BD)_	CT_i (BD)_	AUDPC_i_	CI
NDVI_i (BD)_	1	0.744**	-0.642**	0.282	0.208
CC_i (BD)_		1	-0.383*	0.140	0.061
CT_i (BD)_			1	-0.149	-0.126
AUDPC_i_				1	0.987**
CI					1

*- Significant at p ≤ 0.05, **- Significant at p ≤ 0.01.

**Table 13:** Correlation between vegetation indices and slow rusting parameters during disease under artificial epiphytotic condition.

	NDVI_i (DD)_	CC_i (DD)_	CT_i (DD)_	AUDPC_i_	CI
NDVI_i (DD)_	1	0.688**	-0.418*	-0.624**	-0.648**
CC_i (DD)_		1	-0.386*	-0.585**	-0.585**
CT_i (DD)_			1	0.740**	0.760**
AUDPC_i_				1	0.987**
CI					1

*- Significant at p ≤ 0.05, **- Significant at p ≤ 0.01.

**Table 14:** Correlation between vegetation indices and slow rusting parameters post occurrence of disease.

	NDVI_i (AD)_	CC_i (AD)_	CT_i (AD)_	AUDPC_i_	CI
NDVI_i (AD)_	1	0.790**	-0.675**	-0.455*	-0.471*
CC_i (AD)_		1	-0.605**	-0.496*	-0.537**
CT_i (AD)_			1	0.503*	0.497*
AUDPC_i_				1	0.987**
CI					1

*- Significant at p ≤ 0.05, **- Significant at p ≤ 0.01.

**Table 15:** Correlation between vegetation indices and slow rusting parameters post occurrence of disease with grain yield.

	NDVI_i (AD)_	CC_i (AD)_	CT_i (AD)_	FLA_i_	TGW_i_	AUDPC_i_	CI	PYi
NDVI_i (AD)_	1	0.790**	-0.675**	0.771**	0.412*	-0.455*	-0.471*	0.915**
CC_i (AD)_		1	-0.605**	0.756**	0.437*	-0.496*	-0.537**	0.809**
CT_i (AD)_			1	-0.527**	-0.402*	0.503*	0.497*	-0.632**
FLA_i_				1	0.430*	-0.420*	-0.441*	0.915**
TGW_i_					1	-0.152	-0.172	0.455*
AUDPC_i_						1	0.987**	-0.501*
CI							1	-0.528**
PYi								1

*- Significant at p ≤ 0.05, **- Significant at p ≤ 0.01.

### Correlation among vegetation indices, yield traits and slow rusting parameters

Pearson’s correlation analysis revealed stage-specific relationships among vegetation indices, pathological parameters and yield under disease-inoculated conditions. Prior to disease onset, AUDPC and coefficient of infection (CI) showed no significant association with NDVI, chlorophyll content or canopy temperature, indicating that physiological traits were unaffected at early stages. NDVI was strongly and positively correlated with chlorophyll content (0.744**) and negatively correlated with canopy temperature (−0.642**), reflecting that vigorous, photosynthetically active canopies maintain better thermal regulation. At peak disease, AUDPC and CI exhibited significant negative correlations with NDVI (−0.24**) and chlorophyll content (−0.585**) and a strong positive correlation with canopy temperature (0.740**), demonstrating that increased stripe rust severity reduces greenness and photosynthetic capacity while elevating thermal stress. Similar trends persisted during the declining phase of disease. Yield showed significant positive correlations with NDVI (0.915**), chlorophyll content (0.809**), flag leaf area (0.915**) and thousand grain weight (0.455*), and negative correlations with canopy temperature (−0.632), AUDPC (−0.501) and CI (−0.528*). Collectively, these results confirm that stripe rust severity impairs plant vigor and thermal balance, ultimately reducing grain yield, while healthier canopies contribute to improved productivity (**Tables 12**–**15**).

### Quantitative analysis by linear regression

Linear Regression Analysis was carried out for determination of relationship among AUDPC and various vegetation indices like NDVI, chlorophyll content (CC) and canopy temperature (CT). Analysis revealed that AUDPC is inversely proportional to NDVI (-0.455) and chlorophyll content (-0.496) whereas directly proportional to canopy temperature (0.593).

**Figure 4:**
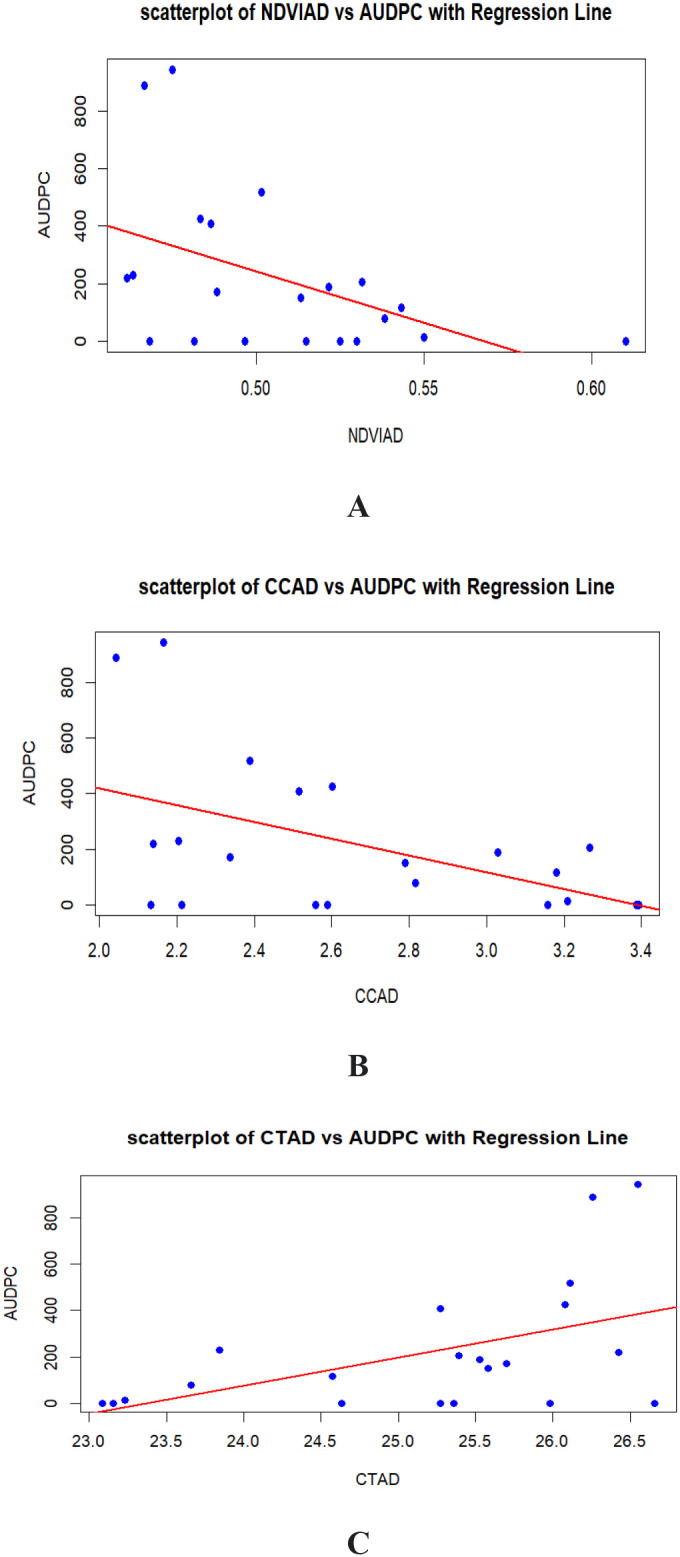
Linear regression analysis **(A)** Relationship between NDVI(AD) and AUDPC, **(B)** Relationship between CC(AD) and AUDPC, **(C)** Relationship between CT(AD) and AUDPC.

The coefficient of determination, or R² value, indicates the proportion of variation in the dependent variable that can be predicted from the independent variable. There is a highly significant linear relationship between NDVI and AUDPC, accounting for 21.79% of the variability. The intercept formula suggests slope value is 2021.9 which means one-unit increase in NDVI results in a decrease in AUDPC by 3558.8 units. Similarly, the relationship between chlorophyll content and AUDPC is highly significant, with 24.67% of the variability explained and response value is -301.2. Chlorophyll content influences AUDPC by reducing its value by 301.2 on each one-unit rise. The relationship between canopy temperature and AUDPC is also highly significant, explaining 25.4% of the variability, and a one-unit increase in canopy temperature raises AUDPC by 121.41 (**Figure 4**).

**Table 16:** Stripe rust resistance marker profiling for *Yr16* and *Yr24* gene in biofortified wheat breeding lines and their parents.

S.No	Advanced breeding lines	Parents of advanced breeding lines with field reaction	*Xgwm102* 150(-), 155,200(+)	*Yr*16	*Barc*181 180(+),220(-)	*Yr*24
1.	JWBL-1	HP-44, (MS)	150,200(-/+)	150,200 (-/+)	220,180(-/+)	180,220,190 (-/+)
		JAUW683, (TR)	155,200(+)		220,180(-/+)	
		HP-45, (0)	155,200(+)		220,180(-/+)	
		HD-3086 (MS)	155,200(+)		180(+)	
2.	JWBL-2	HP-44, (MS)	150,200(-/+)	150,200 (-/+)	220,180(-/+)	180, 220 (-/+)
		RSP-561(S)	155,200(+)		220,200(-)	
3.	JWBL-3	HP-45, (0)	155,200(+)	155,200 (+)	220,180(-/+)	220,180 (-/+)
		JAUW-683, (TR)	155,200(+)		220,180(-/+)	
		HP-44, (MS)	150,200(-/+)		220,180(-/+)	
		HD-3086 (MS)	155,200(+)		180(+)	
4.	JWBL-4	HP-44, (MS)	150,200(-/+)	155,200 (+)	220,180(-/+)	190(-)
		JAUW683, (TR)	155,200(+)		220,180(-/+)	
		HP-45, (0)	155,200(+)		220,180(-/+)	
		HD-3086(MS)	155,200(+)		180(+)	
5.	JWBL-5	RSP-561, (S)	155,200(+)	150,160,210 (-)	220,200(-)	220,190(-)
		HP-25(TR)	150,200(-/+)		220,180(-/+)	
6.	JWBL-6	Longreach Gauntlet, (MR)	150,200(-/+)	150,200 (-/+)	220,190(-)	220,190,180 (-/+)
		HD-3226(TR)	150,200(-/+)		220,180(-/+)	
7.	JWBL-7	HP-44, (MS)	150,200(-/+)	150,200 (-/+)	220,180(-/+)	220,190(-)
		JAUW683, (TR)	155,200(+)		220,180(-/+)	
		HP-45, (0)	155,200(+)		220,180(-/+)	
		HD-3086(MS)	155,200(+)		180(+)	
8.	JWBL-8	HP-25, (TR)	150,200(-/+)	155,200 (+)	220,180(-/+)	220,180, 190(-/+)
		JAUW683, (TR)	155,200(+)		220,180(-/+)	
		Longreach Gauntlet, (MR)	150,200(-/+)		220,190(-)	
		HD-3226 (TR)	150,200(-/+)		220,180(-/+)	
9.	JWBL-9	Longreach Gauntlet, (MR)	150,200(-/+)	150,200 (-/+)	220,190(-)	220,180,190(-/+)
		HD-3226(TR)	150,200(-/+)		220,180(-/+)	
10.	JWBL-10	HP-25, (TR)	150,200(-/+)	150,160,210 (-)	220,180(-)	220,180 (-/+)
		HD-3086(MS)	155,200(+)		180(+)	
11.	JWBL-11	HD-3086, (MS)	155,200(+)	150,200 (-/+)	180(+)	220,180 (-/+)
		HP-25, (TR)	150,200(-/+)		220,180(-/+)	
		RSP-561, (S)	155,200(+)		220,200(-/+)	
		HP-45 (0)	155,200(+)		220,180(-/+)	
12.	JWBL12	JAUW683, (TR)	155,200(+)	150,200 (-/+)	220,180(-/+)	220,180 (-/+)
		HP-25, (TR)	150,200(-/+)		220,180(-/+)	
		HD-3086, (MS)	155,200(+)		180(+)	
		HP-44 (MS)	150,200(-/+)		220,180(-/+)	
13.	JWBL-13	HP-44, (MS)	150,200(-/+)	150,200 (-/+)	220,180(-/+)	220,190,180 (-/+)
		RSP-561, (S)	155,200(+)		220,200(-)	
		HP-44, (MS)	150,200(-/+)		220,180(-/+)	
		HD-3086 (MS)	155(+)		180(+)	

**Figure 5:**
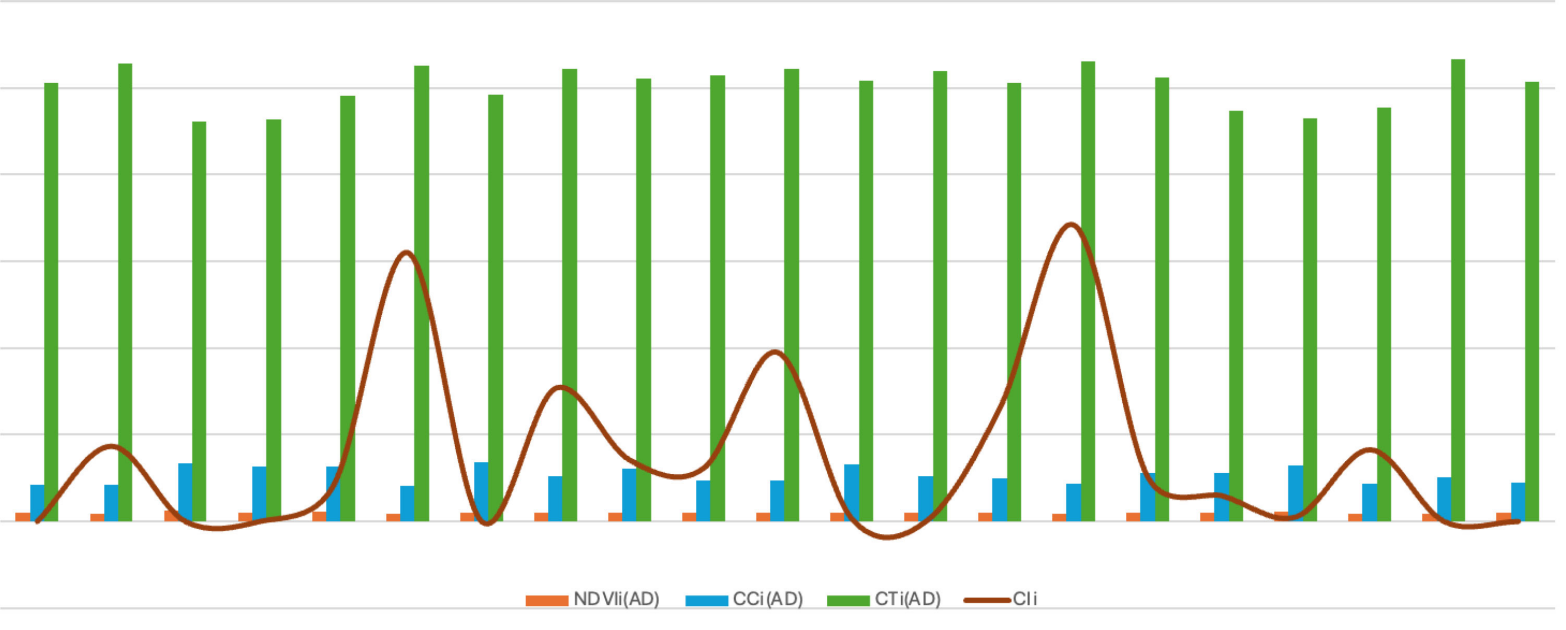
Graphical representation of final disease incidence (FDI) among different genotypes of wheat.

**Figure 6:**
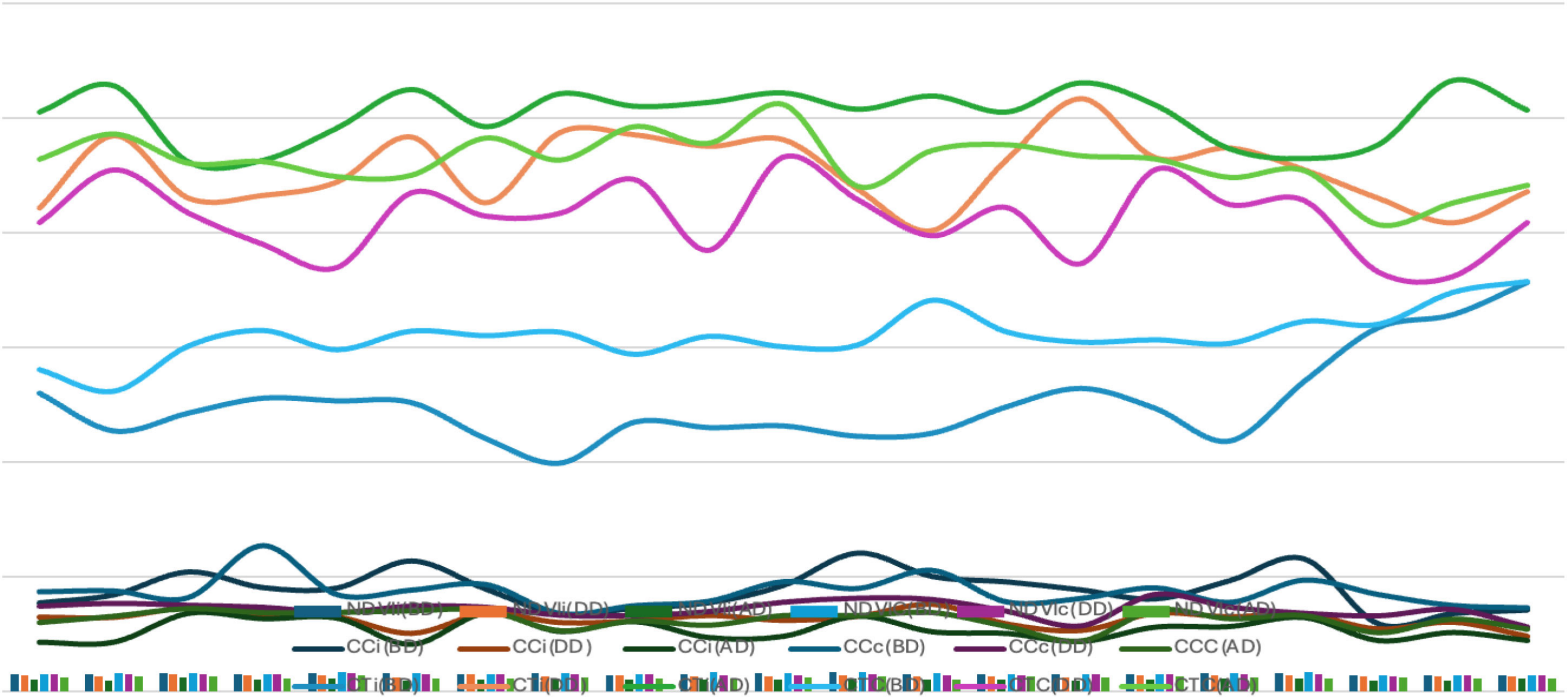
Vegetation indices during different stage of disease under artificial epiphytotic and disease controlled.

Genotypes exhibiting lower FDI consistently maintained higher NDVI and Chlorophyll content with comparatively lower Canopy Temperature, reflecting better physiological resilience. These trends suggest that vegetation indices can serve as reliable, non-destructive indicators for identifying slow rusting or tolerant genotypes in breeding programs (**Figure 5**).

Variation in NDVI, canopy chlorophyll content (CC), and canopy temperature (CT) across wheat genotypes under artificial epiphytotic and controlled conditions. Before disease, values were similar in both conditions, but during disease, NDVI and Chlorophyll content (CC) declined while Canopy Temperature (CT) increased under stress, indicating reduced photosynthetic activity and higher canopy heat load. Genotypic differences were evident, with some maintaining higher NDVI/CC and lower CT under stress, suggesting better tolerance (**Figure 6**).

### Molecular marker profiling for *Yr16* and *Yr24* gene in advanced breeding lines and their parents

Molecular characterization of wheat advanced breeding lines and their parental genotypes have been presented in **Table 16**. The presence of *Yr* genes conferring resistance against stripe rust using twelve SSR markers were utilized to detect the distribution of *Yr* genes in the parental lines, among which two markers (*Xgwm102* and *Barc181*) linked *Yr 16* and *Yr 24* were found to be polymorphic. The presence of two genes, *Yr*16 and *Yr*24 was identified in all parental genotypes, either individually or in combination. Subsequently, these markers were applied to 13 advanced breeding lines to determine their distribution of *Yr* genes.

**Table 17:** Coefficient of determination and correlation coefficient between AUDPC and the three vegetation indices.

Vegetation indices	Coeff. of determination (R^2^)	Correlation coefficient
NDVI	0.2179**	-0.455*
Canopy temperature (CTAD)	0.254**	0.593*
Chlorophyll content (CCAD)	0.2467**	-0.496*

#### Molecular characterization of *Yr*16

**Figure 7:**
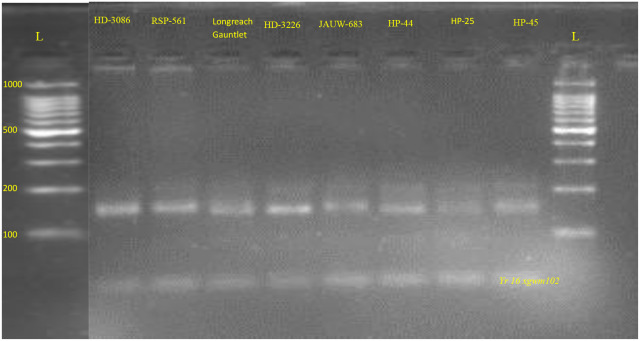
PCR amplification profile of SSR marker *Xgwm102* (*Yr16*) in parental genotypes. *Heterozygous parents, Longreach Gauntlet, HD-3226, HP-44, HP-25 150bp,200bp (-/+); Resistant parent, HD-3086, RSP-561, JAUW-683, HP-45. 155bp,200bp (+).

**Figure 8:**
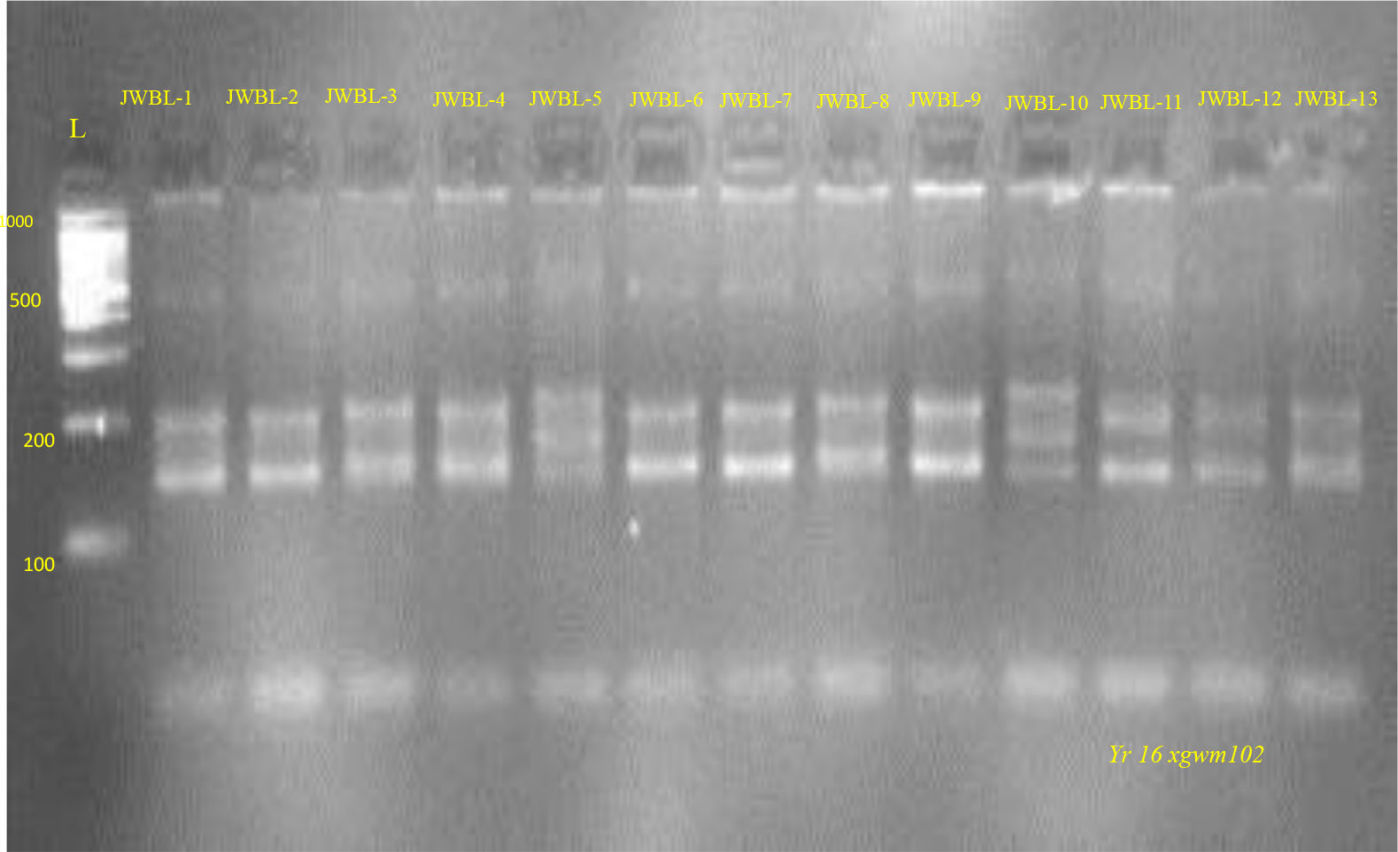
PCR amplification profile of SSR marker *Xgwm102* (*Yr16*) in advanced wheat breading lines. *Heterozygous advanced breeding lines: JWBL-1, JWBL-2, JWBL-5, JWBL-6, JWBL-7, JWBL-9, JWBL-10, JWBL-11, JWBL-12 and JWBL-13 150bp,200bp (-/+). Resistant advanced breeding lines: JWBL-3, JWBL-4, JWBL-8. 155bp,200bp (+). susceptible advanced breeding lines: JWBL-5 AND JWBL-10 150bp (-).

**Figures 7**, **8** represents PCR amplification profile of *Xgwm102* SSR marker which is used to detect the presence of *Yr*16 gene in 21 genotypes which comprises 13 advanced breeding lines and their eight parental genotypes. This marker (*Xgwm102)* amplifies at product size of 150bp (-), 155bp (+) and 200bp (+). *Yr16* was originated from French cultivar *Capelle-Desprez* and shows All Stage Resistance (ASR). Seven genotypes (four parents and three advanced breeding lines) demonstrated the presence of the *Yr*16 gene by amplifying at 155 bp and 200bp (33.33%). These include HD-3086, RSP-561, JAUW-683, HP-45, JWBL3, JWBL-4 and JWBL-8. Fourteen genotypes, comprising four parental lines and ten advanced wheat breeding lines, amplified a product size of 150bp and 200bp (which indicates presence of susceptible and resistant alleles respectively), indicating the presence of the *Yr*16 gene in heterozygous condition (66.66%). These genotypes are Longreach Gauntlet, HD3226, HP-44, HP-25, JWBL-1, JWBL-2, JWBL-6, JWBL-7, JWBL-9, JWBL-11, JWBL-12 and JWBL-13. Two genotypes, JWBL-5 and JWBL-10, showed amplification at a product size of 150bp, 160bp and 210bp (**Table 17**).

#### Molecular characterization of *Yr*24

**Figure 9:**
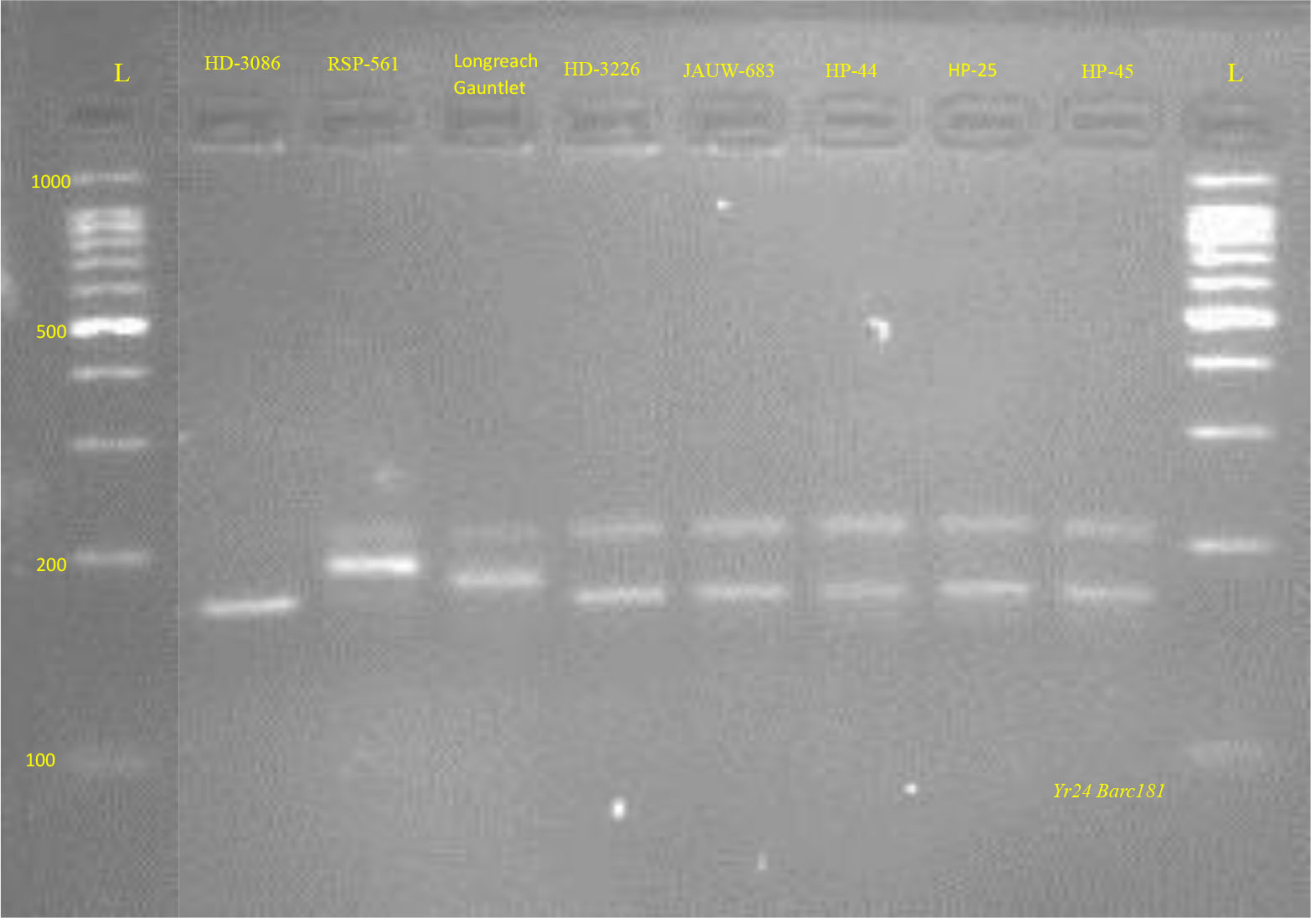
PCR amplification profile SSR marker *Barc181* (*Yr24)* in parental genotypes. *Heterozygous for both susceptible and resistant alleles: HD-3226, JAUW-683, HP-44, HP25, HP-45 220/180bp. Susceptible parents: RSP-561, Longreach Gauntlet 220bp (-). Resistant parents: HD-3086 180bp (+).

**Figure 10:**
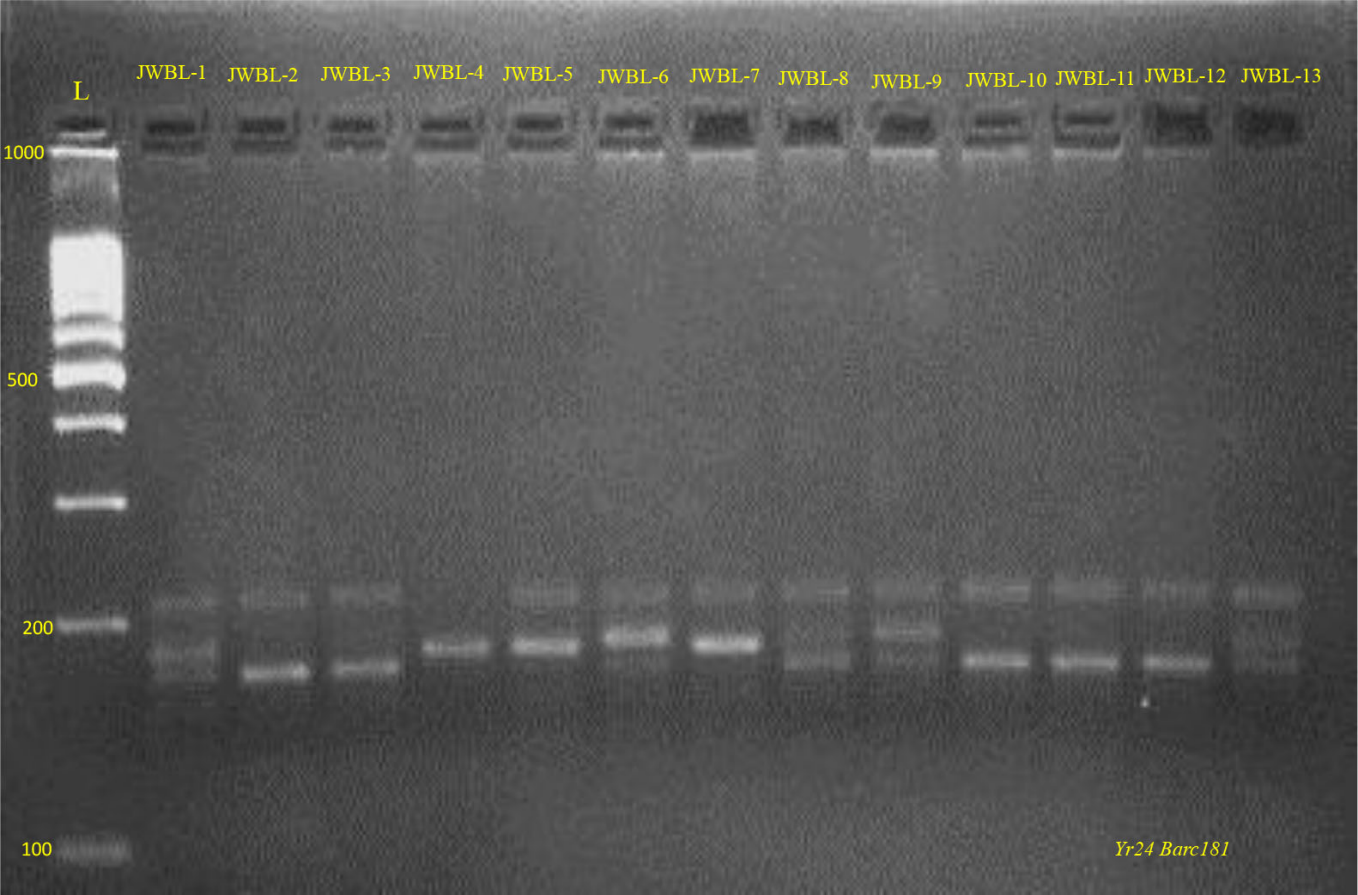
PCR amplification profile of SSR marker *Barc181* (*Yr24*)in advanced wheat breeding lines. *Heterozygous for both susceptible and resistant alleles: JWBL-1, JWBL-2, JWBL-3, JWBL6, JWBL-8, JWBL-9, JWBL-10, JWBL-11, JWBL-12, JWBL-13 220/180 bp (+/-). Susceptible: JWBL-5, JWBL-7180bp (-).

**Figures 9**, **10** represents the PCR amplification profile of *Barc181* SSR marker used to detect the presence of *Yr*24 gene in 21 genotypes and amplifies at 180bp (+) and 220bp (-) product size. This gene conferred All Stage Resistance (ASR) to yellow rust. Ten genotypes JWBL-2, JWBL-3, JWBL-10, JWBL-11, JWBL-12, HD-3226, JAUW-683, HP-44, HP-25 and HP-45 indicated presence of *Yr*24 in heterozygous condition (47.61%). Out of these ten genotypes former five are advanced breeding line while the later are parental genotypes. One parental genotype, HD-3086 amplified at a product size of 180bp confirming the presence of *Yr*24. Five genotypes JWBL-1, JWBL-6, JWBL-8, JWBL-9 and JWBL-13 amplified at 220bp,180bp and 190bp product size indicating the presence of *Yr*24 gene but in heterozygous condition (23.80%). Longreach Gauntlet, JWBL-5 and JWBL-7 amplifies at 220bp and 190bp whereas JWBL-4 amplifies at 190bp. RSP-561 amplifies at 220bp and 200bp.

## Discussion

Recent advancements in wheat breeding, driven by climate change, population growth, changing consumer preferences and improved technologies have significantly influenced breeding objectives. Wheat, one of the earliest domesticated crops, has undergone 10,000 years of selection focused on yield, quality and resistance to biotic stresses (Zhao et al.,2019). However, domestication has reduced genetic diversity, necessitating new approaches to improve yield, biofortification, and resistance to both biotic and abiotic stresses (Wani et al., 2022).

Traditional breeding methods typically takes 8–10 years to develop commercial cultivars rely on inefficient phenotyping. Marker-assisted selection, as highlighted by Jamil et al. (2020) leverages molecular markers to improve selection accuracy and accelerate breeding cycles. The introgression of micronutrient traits into conventional wheat varieties aims at biofortification, now a key breeding objective. These lines are subjected to biotic and abiotic stress screening to identify elite breeding lines with desirable traits. While pyramiding resistance genes can enhance wheat’s resistance to diseases, it also increases the selection pressure on pathogens, potentially leading to super-virulent races. Therefore, the frequent release of new resistant cultivars is crucial.

Innovative screening methods, such as precision phenotyping based on physiological parameters, offer new ways to gather spatial data in precision agriculture (Aarif et al., 2025*)*. Tools like NDVI (Normalized Difference Vegetation Index), first introduced by Kriegler, 1969), absorbance-based chlorophyll content (Yadava, 1986) and infrared radiation-based canopy temperature (Kimes, 1980) allow researchers to assess crop health and field complexity non-destructively (Gao et al. (2024). Stripe rust causing 10-70% yield loss in wheat, as noted by Chen (2005) its early detection is essential. Numerous studies, including those by Bravo et al. (2003); Santin-Janin et al. (2009); Carter and Knapp (2001); Balaghi et al. (2008); Singh et al. (2023) and Atanasov et al. (2025) have demonstrated the relationship between NDVI data from hyperspectral imaging and factors like plant disease, biomass, stress, and yield prediction.

In this study, advanced wheat breeding lines enriched with micronutrients along with their parental lines were evaluated for stripe rust resistance and its impact on vegetation indices and yield. The research utilized marker-assisted selection combined with spectral vegetation indices to accurately correlate disease severity with plant health.

### Genotypic and phenotypic characterization of advanced wheat breeding lines and parental genotypes: analysis of variance and genetic variation

Twenty-one wheat genotypes, including 13 advanced breeding lines and 8 parental lines, were evaluated in a randomized complete block design (RBD) during Rabi 2023-24, with three replications. ANOVA results indicated that vegetation indices, yield parameters and slow rusting traits at different disease development stages were highly significant under both artificial epiphytotic and disease-controlled conditions. Bhatt et al. (2023) similarly found significant genotypic variation for these traits in bread wheat, emphasizing the effectiveness of these lines in responding to disease stress and their potential to enhance yield based on physiological traits.

Genotypic and phenotypic evaluations revealed high heritability and genetic variability for key traits like NDVI, chlorophyll content, canopy temperature, flag leaf area, yield and AUDPC, crucial for breeding programs. NDVI showed moderate to high heritability across disease stages, with close GCV and PCV values, indicating strong genetic control and limited environmental influence. These findings are consistent with Naveen et al. (2023), who reported effective selection based on phenotypic variation when GCV and PCV differences were minimal. Traits like chlorophyll content and canopy temperature showed moderate to high heritability with significant genetic advance particularly in disease stages indicating their potential for selection. In contrast, traits like 1000-grain weight despite having high heritability exhibited low genetic advance and suggested limited improvement potential because of low additive variance. Plot yield and AUDPC are important parameters for commercial breeding, showed high heritability and genetic advance under disease-controlled conditions underscoring their breeding significance. These results align with findings from Saeed et al. (2022), who reported high heritability for traits like total chlorophyll and NDVI in wheat genotypes, further validating the importance of these traits for selection.

### T-test analysis of stripe rust impact on advanced breeding lines and parental genotypes

T-test analysis revealed significant differences in all parameters between epiphytotic and disease-controlled conditions across various disease stages. Traits such as NDVI, chlorophyll content, flag leaf area, 1000-grain weight and yield were higher in disease-free environments while canopy temperature was elevated in diseased plants due to reduced transpiration efficiency. These results highlight the critical impact of disease on physiological and morphological traits and emphasize the importance of disease control for optimizing yield and crop performance.

Genetic diversity among the breeding lines, initially developed for biofortification with traits like zinc and iron, likely contributed to varying stripe rust resistance. Independent t-tests confirmed significant differences between disease and control conditions for most traits, supporting the effectiveness of this approach in differentiating responses to disease stress. Yuan et al. (2014) similarly used t-tests to distinguish between yellow rust and powdery mildew infections. The combination of statistical methods and physiological indices offers a comprehensive approach to disease management and breeding for disease resistance.

Vegetation indices and yield traits showed low coefficients of variation (CVs), indicating consistent and reliable measurements, while the disease progression curve had a higher CV, reflecting significant variability in disease development. This variability could be attributed to genetic differences among genotypes and environmental factors, underscoring the need for robust disease resistance strategies in breeding programs.

### Field-based screening and slow rusting parameters

The field-based screening of advanced wheat breeding lines and their parental genotypes at the Experimental Area, Division of Plant Breeding & Genetics, Chatha (Rabi 2024) demonstrated significant variability in resistance to stripe rust. The genotypes were categorized into six groups, from Resistant (R) to Susceptible (S). Lines such as JWBL-1, JWBL-3, JWBL-4, JWBL-7, JWBL-12, JWBL-13, JAUW-683, HP-25, and HP-45 showed high resistance with minimal or no infection. Moderately resistant lines included Longreach Gauntlet, while lines like JWBL-5, HD-3226, and others exhibited varying responses. Slow rusting parameters, such as Coefficient of Infection (CI), Coefficient of Disease Level (CDL), Area Under Disease Progress Curve (AUDPC) and Final Rust Severity (FRS), were also evaluated. Resistant lines showed low values for these metrics, while susceptible lines like RSP-561, JWBL-6 and JWBL-11 had higher values indicating more severe infections. JWBL3, JWBL-12, and JWBL-13 were identified as promising lines for further development into resistant wheat varieties.

### Correlation among different vegetation indices, yield traits and disease parameters

Pearson’s Correlation analysis revealed crucial insights into the relationships between vegetation indices, yield traits, and pathological parameters under disease-inoculated conditions. Prior to disease onset, the lack of significant correlation between AUDPC/coefficient of infection and NDVI, chlorophyll content, or canopy temperature suggests initial plant health is not yet compromised by disease factors. However, the strong positive correlation between NDVI and chlorophyll content (0.744**) and the negative correlation between NDVI and canopy temperature (-0.642**) highlight the integral role of plant Vigor and thermal regulation in early disease resistance.

During peak disease stages, the significant negative correlations of AUDPC and CI with NDVI (-0.24**), chlorophyll content (-0.585**), and their positive correlation with canopy temperature (0.740**) indicate that severe disease progression leads to decreased plant health and increased thermal stress. As the disease wanes, the negative correlations of AUDPC with NDVI (-0.455*) and chlorophyll content (-0.496*), alongside the positive correlation with canopy temperature (0.503*), further emphasize the adverse effects of disease on plant vitality and thermal management. stripe rust significantly impacts the relationship between canopy temperature and chlorophyll content through the process of evapotranspiration. During the peak of the disease, the negative correlation between chlorophyll content and canopy temperature (-0.675**) indicates that as the disease severity increases, chlorophyll content decreases while canopy temperature rises. Evapo-transpiration, the combined process of water evaporation from soil and plant surfaces and transpiration from plant leaves, plays a crucial role here. Healthy plants with high chlorophyll content typically exhibit higher rates of transpiration, which helps cool the canopy by releasing water vapor. However, stripe rust damages the plant’s photosynthetic apparatus, reducing chlorophyll content and thereby decreasing transpiration rates. Consequently, the reduced transpiration leads to higher canopy temperatures. This elevated temperature further stresses the plant, exacerbating the negative impact of the disease and creating a feedback loop that hinders the plant’s ability to maintain optimal temperature and water balance. Understanding this relationship is vital for developing effective disease management strategies to protect crop health and yield. However, the highest disease development was observed within the temperature range of 18 to 28 °C. higher the canopy temperature lesser will be the plant health and chlorophyll content.

Furthermore, the significant positive correlations of plot yield with NDVI (0.915**), chlorophyll content (0.809**), flag leaf area (0.915**), and thousand grain weight (0.455*) underscore the importance of robust plant health, photosynthetic capacity, and structural traits in maximizing yield. Conversely, the negative correlations of plot yield with canopy temperature (-0.632), AUDPC (-0.501), and the coefficient of infection (-0.528*) illustrate the detrimental impact of disease and thermal stress on yield outcomes. Kumar et al. (2024) found that grain yield exhibited a positive and significant correlation with several factors, including NDVI, chlorophyll index, flag leaf length, flag leaf area, tiller per plant, number of grains per spike, peduncle length, and 1000 grain weight (TGW). This pattern was observed under heat stress during the grain-filling period (GFD) and in the presence of rust diseases and spot blotch in wheat. Arora et al. (2019) also found a significant correlation between AUDPC scores and NDVI values, suggesting that this method could be useful for rust pathologists. These findings elucidate the complex interplay between disease dynamics, plant health, and yield components, reinforcing the critical need for integrated disease management strategies to sustain high yield and plant resilience in the face of stripe rust challenges.

### Linear regression

The linear regression analysis between AUDPC (Area Under Disease Progress Curve) and various vegetation indices—NDVI, chlorophyll content, and canopy temperature—reveals significant correlations that highlight the impact of disease on plant health. NDVI and chlorophyll content show significant negative correlations with AUDPC (r = -0.455 and r = -0.496, respectively), indicating that increased disease severity leads to reductions in plant greenness and photosynthetic capacity, with 21.79% and 24.67% of their variability explained by AUDPC. Conversely, canopy temperature exhibits a positive correlation with AUDPC (r = 0.593), explaining 25.4% of its variability, suggesting that disease stress increases plant metabolic activity and thermal stress. This relationship likely reflects the physiological stress experienced by plants under disease pressure, resulting in increased respiration rates and reduced transpiration cooling, which collectively elevate canopy temperature. Monitoring canopy temperature, therefore, provides a valuable indicator of plant stress and disease impact. The coefficient of infection (CI) has a nearly perfect positive correlation with AUDPC (r = 0.987), accounting for 97.46% of its variability, underscoring AUDPC’s reliability as a measure of disease impact. This strong relationship underscores the utility of AUDPC in predicting and quantifying the extent of disease impact, making it a critical tool for researchers and pathologists. These findings collectively emphasize the importance of integrating vegetation indices into disease monitoring and management frameworks. NDVI, chlorophyll content, and canopy temperature serve as valuable proxies for early detection and quantification of disease stress, facilitating timely and effective interventions. Arora et al. (2014) also employed simple linear regression analysis to investigate the relationship between AUDPC and three indices in various wheat varieties. They observed similar trends and regression equations for AUDPC in relation to NDVI, chlorophyll content, and canopy temperature. The results of his study have confirmed the use of spectral canopy indices as effective tool for quantifying damage caused by stripe rust in terms of precision but also can be used as a new approach to save time and money.

### Validation of molecular characterization of advanced breeding lines for *Yr* genes

The molecular characterization of wheat advanced breeding lines, biofortified for Zn and Fe and their parental genotypes aimed to detect effective *Yr* genes for resistance to stripe rust. SSR markers were used to identify *Yr* genes. The *Xgwm102* marker revealed the presence of the *Yr*16 gene in four parental genotypes (HD-3086, RSP-561, JAUW-683, HP-45) and 11 advanced breeding lines, though some showed heterozygosity for *Yr*16. Rani et al. (2019) confirmed the presence of *Yr*16 in 22 genotypes by amplifying at 155 and 200 bp. Similarly, the *Barc181* marker identified the *Yr*24 gene, with 10 genotypes heterozygous for the gene. Two genotypes (JWBL-5 and JWBL-7) lacked *Yr*24, while HD-3086 confirmed its presence.

Haider et al. (2023) confirmed the SSR marker *Barc*181 is linked to the *Yr*24 gene at 6.7 cM, which amplified two types of alleles: 180 bp, signifying the presence of *Yr*24, and 200 bp, indicating its absence. These findings highlight the potential for incorporating these *Yr* genes into breeding programs for developing rust-resistant, nutrient-enriched wheat varieties, enhancing both disease resistance and nutritional quality. The use of SSR markers for molecular characterization supports the development of superior wheat cultivars essential for sustainable agriculture and food security.

## Conclusion

The effectiveness of integrating genomic tools and remote sensing-based vegetation indices for precision phenotyping of stripe rust resistance in advanced wheat lines is elucidated. Significant genetic variability among the 21 genotypes under both artificial epiphytotic and controlled conditions emphasizes the importance of combining phenotypic and genotypic data for informed selection.

High heritability in traits such as NDVI, chlorophyll content and canopy temperature suggests their utility as reliable indicators for early disease detection and selection under biotic stress. Molecular marker analysis successfully identified *Yr* resistance genes in several genotypes, with JWBL-3, JWBL-12, and JWBL-13 emerging as promising candidates with stable resistance and favourable agronomic traits.

Dual screening with molecular markers and spectral phenotyping, this approach enhances selection accuracy and efficiency, offering a scalable, data-driven framework for the development of stripe rust-resistant, nutrient-rich, and high-yielding wheat varieties. The integration of genomics and remote sensing paves the way for next-generation breeding strategies focused on sustainability and resilience in wheat improvement programs.

## Data Availability

The datasets presented in this study can be found in online repositories. The names of the repository/repositories and accession number(s) can be found in the article/supplementary material.
